# Rapid Effects of Vagus Nerve Stimulation on Sensory Processing Through Activation of Neuromodulatory Systems

**DOI:** 10.3389/fnins.2022.922424

**Published:** 2022-07-05

**Authors:** Charles Rodenkirch, Jason B. Carmel, Qi Wang

**Affiliations:** ^1^Department of Biomedical Engineering, Columbia University, New York, NY, United States; ^2^Jacobs Technion-Cornell Institute, Cornell Tech, New York, NY, United States; ^3^Department of Neurology and Orthopedics, Columbia University Medical Center, New York, NY, United States

**Keywords:** vagus nerve stimulation, sensory processing, neuromodulation, cholinergic system, dopaminergic system, serotonergic system, locus coereleus, noradrenergic system

## Abstract

After sensory information is encoded into neural signals at the periphery, it is processed through multiple brain regions before perception occurs (i.e., sensory processing). Recent work has begun to tease apart how neuromodulatory systems influence sensory processing. Vagus nerve stimulation (VNS) is well-known as an effective and safe method of activating neuromodulatory systems. There is a growing body of studies confirming VNS has immediate effects on sensory processing across multiple sensory modalities. These immediate effects of VNS on sensory processing are distinct from the more well-documented method of inducing lasting neuroplastic changes to the sensory pathways through repeatedly delivering a brief VNS burst paired with a sensory stimulus. Immediate effects occur upon VNS onset, often disappear upon VNS offset, and the modulation is present for all sensory stimuli. Conversely, the neuroplastic effect of pairing sub-second bursts of VNS with a sensory stimulus alters sensory processing only after multiple pairing sessions, this alteration remains after cessation of pairing sessions, and the alteration selectively affects the response properties of neurons encoding the specific paired sensory stimulus. Here, we call attention to the immediate effects VNS has on sensory processing. This review discusses existing studies on this topic, provides an overview of the underlying neuromodulatory systems that likely play a role, and briefly explores the potential translational applications of using VNS to rapidly regulate sensory processing.

## Introduction

Accurate and detailed perception of tactile, auditory, and visual stimuli is critical for completing a large variety of tasks, including many necessary for daily life and independent living. Perceptual acuity is dependent upon both reliable transduction of sensory stimuli into neural signals at the periphery and high-fidelity processing of sensory information by the central nervous system. Once sensory information is transduced into neural activity by sensory receptors, it is processed through multiple stages of the sensory pathway before perception occurs (i.e., central sensory processing) (Wall and Dubner, 1972; Rodieck, 1979; Reid and Alonso, 1995; Chechik et al., 2006; Wang et al., 2010; Ollerenshaw et al., 2012). Developing methods that use neuromodulation of sensory processing to improve sensory acuity is of great interest as many significant clinical, commercial, and consumer problems stem from misperception or miscommunication. A growing body of evidence strongly suggests that vagus nerve stimulation (VNS) is a safe and effective method of neuromodulation (Collins et al., 2021). In this mini-review, we explore the effects of VNS on sensory processing. Multiple recent reviews have discussed in detail the ability of short VNS bursts repeatedly paired with sensory stimuli to catalyze neuroplastic reorganization of sensory pathways after multiple pairing sessions (Engineer et al., 2013; Hays, 2016; Engineer, 2019), likely *via* engagement of neuromodulatory systems including the acetylcholine system (Kilgard and Merzenich, 1998). Here, we instead specifically call attention to the immediate effects VNS has on sensory processing and discuss how they likely arise from VNS activating neuromodulatory systems that innervate sensory processing pathways.

Sensory processing is highly dependent upon behavioral states such as attention and arousal (Nicolelis and Fanselow, 2002; Carrasco et al., 2004; Niell and Stryker, 2010; Bennett et al., 2013; Reimer et al., 2014, 2016; McGinley et al., 2015; Vinck et al., 2015; Zheng et al., 2015; Schriver et al., 2018, 2020; Liu et al., 2021) as both are heavily influenced by the same global neuromodulatory systems, including the noradrenergic (Berridge and Waterhouse, 2003; Aston-Jones and Waterhouse, 2016; Liu et al., 2017; Chandler et al., 2019) and cholinergic systems (Pinto et al., 2013). For example, our laboratory recently demonstrated that activation of the locus coeruleus – norepinephrine system (LC-NE), a major neuromodulator of attention and arousal, rapidly enhanced somatosensory processing through NE-mediated suppression of burst spiking induced by calcium T-type channels (Rodenkirch et al., 2019). This NE-enhanced sensory processing increased accuracy of encoded information and improved perceptual sensitivity of awake rats performing tactile discrimination tasks.

## Lasting Alterations to Sensory Processing Occur Over Time When a Sensory Stimulus is Repeatedly Paired With Phasic Vagus Nerve Stimulation

A large body of previous work has focused on using a short burst of VNS repeatedly paired with a brief sensory stimulus to induce reorganization of sensory pathways. This work was inspired by studies which found pairing an auditory tone with phasic activation of dopaminergic, cholinergic, or noradrenergic neuromodulatory systems resulted in a lasting shift of frequency selectivity for neurons in the auditory cortex that selectively favors the paired tone’s frequency (Kilgard and Merzenich, 1998; Bao et al., 2001; Nichols et al., 2011; Martins and Froemke, 2015). We will not review these studies in detail here as they have already been well reviewed previously (Engineer et al., 2013; Hays, 2016; Engineer, 2019).

In general, these studies have delivered phasic VNS (e.g., 0.5 s, 30 Hz, 0.8 mA, 100 μs biphasic pulses) in pair with a specific sensory stimulus (e.g., a specific frequency auditory tone or tactile tap) repeatedly across multiple sessions (e.g., 300 times/day, 20 days). This alters sensory processing in a manner that facilitates detection of the specific paired stimulus (Martinez-Vargas et al., 2009; Engineer et al., 2011; Meyers et al., 2019; Darrow et al., 2020; Lai and David, 2021) and accordingly disfavors detection of non-paired stimuli. This mechanism of action can be strengthened over multiple sessions of pairing to produce long-term permanent reorganization of sensory pathways that alters perception. Taken together, these works suggest phasic VNS has great potential as a next generation neuromodulation technology for rehabilitative motor and sensory therapies (Neuhaus et al., 2007; Kreuzer et al., 2014; Engineer et al., 2015; Tyler et al., 2017; Vanneste et al., 2017; Kilgard et al., 2018; Adcock et al., 2020; Llanos et al., 2020; Thakkar et al., 2020; Altidor et al., 2021; Phillips et al., 2021).

## Transient Modulation of Sensory Processing Occurs Rapidly Upon Vagus Nerve Stimulation Onset

The purpose of this review is to bring light to recent studies indicating VNS modulates sensory processing immediately upon onset. Here, we will discuss in detail studies investigating the immediate effects VNS has on the response properties of neurons along the sensory pathways.

### Tonic Vagus Nerve Stimulation Drives a Rapid and Transient Enhancement of Tactile Processing

**FIGURE 1:**
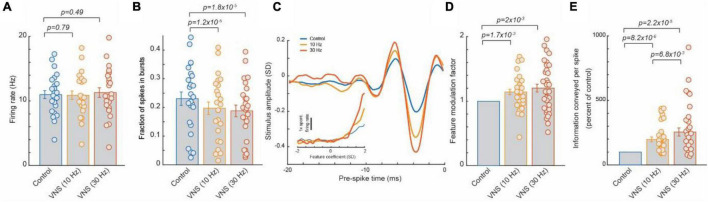
Tonic VNS suppressed burst spiking of thalamocortical neurons and increased the selectivity of their response to the specific stimulus feature they encode, leading to a greater amount of sensory-related information transmitted. **(A)** VNS did not significantly alter firing rate of ventral posteromedial nucleus (VPm) neurons responding to white gaussian noise whisker (WGN) stimulation. **(B)** VNS reduced likelihood of VPm burst spikes, multiple successive spikes with a short inter-spike-intervals (∼4 ms or less) commonly occurring after an extended period of quiescence (∼100 ms) due to calcium t-channel current. **(C,D)** The amplitude of the specific kinetic feature(s) (i.e., whisker deflection) each VPm neuron was selective for was much larger when recovered during VNS, indicating VNS increased selectivity of response. **(E)** Enhanced feature selectivity of VPm neurons during VNS results in a significant increase in amount of the sensory-related information transmitted per spike. Adopted from Rodenkirch and Wang (2020).

Our laboratory has recently demonstrated that VNS can be used to induce a rapid, general improvement of thalamic sensory processing (Figure 1). This is a continuation of our team’s studies investigating the effects of the LC-NE system on thalamocortical circuitry (Rodenkirch et al., 2019), a critical stage for sensory processing and perception (Saalmann and Kastner, 2011; Stanley et al., 2012; Wang et al., 2012; Millard et al., 2013; Kelly et al., 2014; Ollerenshaw et al., 2014; Wimmer et al., 2015; Rikhye et al., 2018). These studies found that direct activation of the LC-NE system (electrical or optogenetic), in a continuous tonic fashion, optimized intrathalamic dynamics for sensory processing. Specifically, tonic LC stimulation (continuous, 5 Hz, 60 μA, 500 μs biphasic pulses) increased the efficiency and rate of sensory-related information transmitted by thalamocortical neurons (Rodenkirch et al., 2019). Further, the observed NE-enhancement of sensory processing resulted in a significant improvement in perceptual sensitivity for rats tasked with discriminating between whisker stimuli of different frequencies. Through pharmacological manipulation it was determined that tonic LC activation improved thalamic sensory processing because a steady increase in NE concentration precludes priming, and in turn activation, of thalamic T-type calcium channels. When active, T-type calcium channels introduced a non-linear bursting response that degraded transmission of detailed sensory information.

Vagus nerve stimulation has been shown to activate the LC-NE system (Hulsey et al., 2017) and is accessible in a non-invasive manner, unlike the LC deep in the brainstem. Therefore, our team next investigated whether tonic VNS would drive similar rapid beneficial effects on sensory processing. Through testing the effects of multiple patterns of VNS on sensory processing, the beneficial effect was found to be highly transient (i.e., benefit begins to dissipate within seconds of ceasing VNS) (Rodenkirch and Wang, 2020). For example, duty-cycled VNS (30 s on/60 s off duty cycle, 30 Hz, 500 μs biphasic pulses) enhanced tactile sensory processing during the on cycle, but this enhancement rapidly dissipated during the off cycle, suggesting that cycling VNS on and off creates fluctuations in sensory processing that would likely be sub-optimal for discrimination. This indicated that an uninterrupted pattern is required to produce a stable benefit. Indeed, continuous tonic VNS (continuous, 30 Hz, 500 μs biphasic pulses) induced a steady enhancement of sensory processing similar to that observed with direct tonic LC stimulation. This immediate enhancement of sensory processing during continuous, tonic VNS was found to be reliably present across recorded neurons. As each recorded neuron encoded for a unique kinetic feature of the whisker stimuli, this suggests the tonic VNS modulation provided a general enhancement of sensory processing regardless of stimulus input. This effect is distinct relative to the selective facilitation of responses to a specific sensory stimulus found after repeatedly pairing VNS bursts with that sensory stimulus.

Further, testing of various tonic VNS current levels and frequencies showed the beneficial effect of tonic VNS on sensory processing increased with intensity and frequency (10 vs. 30 Hz, 0.4 vs. 1 and 1.6 mA) and did not exhibit the inverted U-shape function of effect strength that has been observed with other types of VNS modulation (Morrison et al., 2019) (at least within the parameter ranges tested).

### Vagus Nerve Stimulation Has Rapid Effects on Evoked Responses in the Auditory Cortex

Other research groups working with human subjects have published findings that suggest VNS has immediate beneficial effects on auditory processing. One study in humans who had been receiving chronic VNS (*via* implanted cuffs as a treatment for epilepsy), found VNS enhanced performance on a standard auditory oddball task when compared to performance after their VNS device was turned off (De Taeye et al., 2014). Specifically, during VNS (7 s on/18 s off duty cycle, 20–30 Hz, 0.75–3 mA, 250–500 μs pulses) both accuracy and response time were improved for participants tasked with responding to low frequency target audio tones while ignoring high frequency non-target tones. This same study analyzed auditory event-related potentials (AERP), measured *via* EEG, and found that during VNS, AERP amplitude was also increased. However, the effect on AERP was only significant in individuals whose epilepsy symptoms had positively responded to VNS treatment. A separate study investigating transcutaneous auricular vagus nerve stimulation (taVNS) (30 s on/30 s off duty cycle, 25 Hz, 250 μs pulses) in healthy adults found similar results. Specifically, taVNS increased the strength of AERPs during an oddball auditory task (Rufener et al., 2018). As this study used low frequency tones as non-targets and high frequency tones as targets, a reversal of the prior discussed oddball auditory task, taken together they suggest immediate VNS modulation of auditory response is not specific to low or high frequency audio tones. Another study delivering continuous taVNS (25 Hz, 500 μs biphasic pulses) to healthy adults analyzed the neural response to auditory tones using magnetoencephalography (MEG) instead of EEG and found taVNS altered synchrony of brain activity (Hyvarinen et al., 2015). Further, recent studies using fMRI to monitor neural activity have shown taVNS rapidly affects auditory processing pathways. When taVNS (25 Hz, 0.1 to 1.8 mA, 500 μs monophasic pulses) was delivered to male adults with chronic tinnitus, fMRI recordings exhibited altered activity of multiple brain regions involved with auditory processing (Yakunina et al., 2018). More recently, analysis of fMRI data from human subjects receiving taVNS indicated increased activity in the thalamus and auditory cortex (Peng et al., 2018), suggesting VNS rapidly modulates central auditory sensory processing in humans.

These findings in humans are further supported by multiple electrophysiological and behavioral work in animals that found VNS rapidly affects the response properties of neurons of the auditory pathway. In isoflurane-anesthetized rats, the responses of neurons along the auditory pathway were compared with and without VNS delivered *via* an implanted VNS cuff (30 s on/5 min off duty cycle, 10 Hz, 0.5 mA, 130 μs pulses). The baseline condition was recorded without any ongoing VNS. The VNS condition consisted of discontinuous duty-cycled VNS where auditory testing was performed only during the off periods of the VNS duty cycle. Here they found duty-cycled VNS weakened stimulus-specific adaptation in the cortex but not the thalamus (Shiramatsu et al., 2016), suggesting VNS may modulate thalamocortical transmission but not earlier stages of the auditory pathway. Further work by the same group, using the same paradigm, found VNS predominantly increased the amplitudes of auditory-evoked potentials in the sensory cortex (Takahashi et al., 2020).

### Vagus Nerve Stimulation Modulates Olfactory and Gustatory Processing

The immediate effects of VNS on olfactory processing had been demonstrated as early as the 1980s. Specifically, a study in rats found that a single pulse of VNS from an implanted cuff (0.8–1.5 mA, 200 μs monophasic pulses) reliably evoked firing in the homolateral olfactory bulb (HOB) (Garcia-Diaz et al., 1984). Further evidence that VNS affects olfactory processing was found in more recent studies that used positron emission tomography (PET) to analyze the effects of VNS in awake rats. A PET scan conducted during the time period when the VNS cuff was switched on for the first time (30 s on/5 min off duty cycle, 30 Hz, 1.5 mA, 500 μs pulses) found VNS induced a significant increase in glucose metabolism in both olfactory bulbs (Dedeurwaerdere et al., 2005). However, another study in humans with implanted VNS cuffs for treatment of depression found that whether VNS (30 s on/5 min off duty cycle, 20 Hz, 1.25 mA) was on or off had no effect on subjects’ ability to discriminate or detect olfactory stimuli (Sperling et al., 2011). Yet that same study did find that VNS significantly increased the intensity of the taste of sweet and bitter, suggesting that VNS may rapidly affect gustatory processing as well.

## Vagus Nerve Stimulation Activates Multiple Neuromodulatory Systems That Rapidly Influence the Response Properties of Neurons Along Sensory Pathways

**FIGURE 2:**
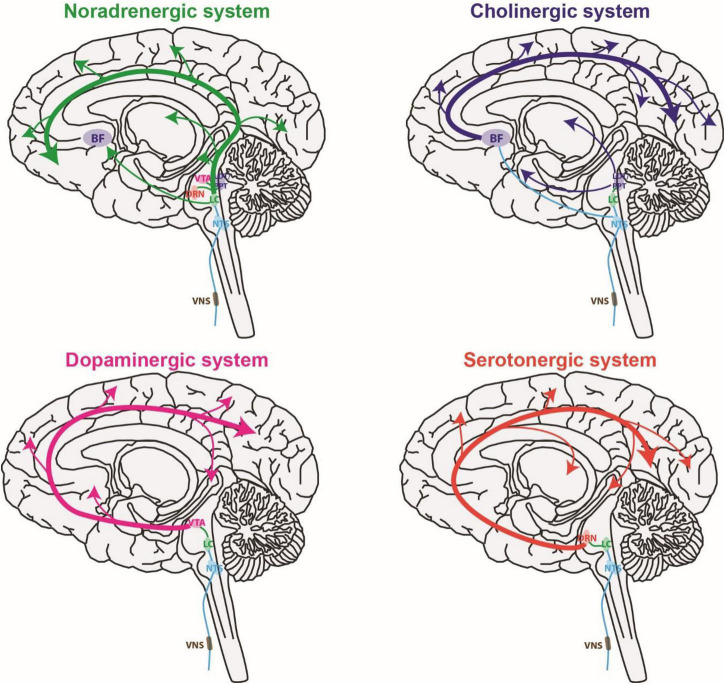
Vagus nerve stimulation activates multiple global neuromodulatory systems that are known to influence sensory processing. BF, basal forebrain; DRN, dorsal raphe nucleus; LC, locus coeruleus; LDT, laterodorsal tegmental nucleus; NTS, nucleus tractus solitaries; PPT, pedunculopontine tegmental nucleus; VTA, ventral tegmental area.

The ability of VNS to have immediate effects on sensory processing is likely due to VNS activating neuromodulatory systems (Figure 2). Here we briefly review studies of the effect of VNS on neuromodulatory systems in both human and animal models. Neurons in the neuromodulatory systems and sensory pathways discussed here can exhibit either tonic or burst spiking patterns (McCormick and Prince, 1988; Nuñez, 1996; Ramcharan et al., 2000; Devilbiss and Waterhouse, 2011; Rodenkirch et al., 2019). Tonic spiking refers to sustained firing of individual spikes at relatively slow rates compared to phasic. Phasic spiking refers to transient bursts of multiple spikes with short inter-spike-intervals. For neuromodulatory systems, the rate of continuous tonic spiking modulates brain state (e.g., attention and arousal) whereas phasic firing is linked with events (e.g., reward, sensory stimuli, and decision-making) and thought to regulate learning and behavior (Rajkowski et al., 1994; Parikh and Sarter, 2008). For sensory pathways, tonic encoding is favored during periods of increased attention and is thought to be more optimal for discrimination of sensory detail (Sherman, 2001a; Rodenkirch et al., 2019). Conversely, bursting responses to sensory stimuli are more likely when drowsy or inattentive and provide a strong encoding that facilitates detection, potentially serving as a wake-up call (Swadlow and Gusev, 2001; Weyand et al., 2001). It is important to note that neuromodulatory systems are well preserved over evolution, and the function of neuromodulatory systems are similar in humans and other mammals such as rodents (Avery and Krichmar, 2017). Indeed, the studies discussed earlier confirm VNS affects sensory processing in both rodents and humans.

### Vagus Nerve Stimulation and the Noradrenergic System

The LC is the primary source of NE in the forebrain (Sara, 2009). The LC exhibits constant tonic firing (1–5 Hz) that regulates brain state (e.g., arousal) as well as intermediate phasic burst spiking events (2–5 spikes at 10–20 Hz per burst) that occur in response to salient sensory stimuli as well as when decisions or responses are made (Devilbiss and Waterhouse, 2011). These two firing modes have been shown to produce distinctly different modulations of the response properties of sensory neurons (Devilbiss and Waterhouse, 2011). The LC innervates multiple regions along the sensory pathway, including the sensory thalamus and cortex (Morrison and Foote, 1986; Simpson et al., 1997).

There is a large body of evidence showing that the LC-NE system modulates sensory processing and perceptual learning (Manunta and Edeline, 1997; Ego-Stengel et al., 2002; Hirata et al., 2006; Doucette et al., 2007; Martins and Froemke, 2015; McBurney-Lin et al., 2019; Waterhouse and Navarra, 2019). Moreover, it is well documented that activation of the LC-NE system immediately modulates the response of sensory neurons. *In vitro*, NE has a depolarizing effect on auditory and visual thalamic relay neurons that coincides with a suppression of burst spiking (McCormick and Prince, 1988). This likely occurs because NE depolarization prevents the extended hyperpolarized periods needed to prime the calcium T-type channels responsible for bursts (Sherman, 2001a). *In vivo*, tonic LC activation has been found to reduce spontaneous activity of the somatosensory thalamus, while facilitating sensory evoked activity, resulting in an increase in signal to noise ratio (Hirata et al., 2006). Our team has shown how tonic LC-NE activation enhances the accuracy of encoded stimuli in the somatosensory thalamus by reducing the fluctuating influence of the calcium T-type channels responsible for bursting. Within the cortex, the LC-NE system can cause either facilitation or inhibition with resulting effect specific to the sensory modality, cell, and stimulation pattern (Devilbiss and Waterhouse, 2004; Videen et al., 1984; Sato et al., 1989; Vazey et al., 2018).

Vagus nerve stimulation’s ability to activate the LC-NE system has long been hypothesized to underlie, in part, the clinical benefits of VNS (Slater and Wang, 2021). VNS is thought to activate the LC *via* the vagus nerve’s afferent projections to the nucleus tractus solitarius (NTS) (Van Bockstaele et al., 1999; Ruffoli et al., 2011). The NTS then sends an excitatory signal to the LC, likely *via* the nucleus paragigantocellularis (Ennis and Aston-Jones, 1988; Reyes and Van Bockstaele, 2006). Indeed, multiple studies have confirmed VNS readily activates the LC-NE system in both animals and humans. In rats, VNS delivered *via* an implanted cuff has been shown to increase the activity of LC neurons as confirmed by electrophysiological recordings under halothane (Groves et al., 2005), chloral hydrate (Dorr and Debonnel, 2006), equithesin (Manta et al., 2009a), and ketamine (Hulsey et al., 2017) as well as by immunohistochemical biomarkers of short-term neuronal activation (Cunningham et al., 2008). Similarly, multiple studies have found that microdialysis samples taken from rats receiving VNS exhibited increased NE concentration in the primary hippocampus (Raedt et al., 2011), basolateral amygdala (Hassert et al., 2004), and cortex (Roosevelt et al., 2006; Follesa et al., 2007; Manta et al., 2013). Finally, the findings in animals seem to be conserved in humans, as fMRI data from a study of adult males with tinnitus indicated taVNS activates the NTS and LC (Yakunina et al., 2018). However, variations in VNS parameters may affect how reliably VNS drives the LC-NE system, as one study measuring NE concentration in the CSF of patients receiving VNS as a treatment for depression failed to detect a significant change (Carpenter et al., 2004).

In addition to direct evidence VNS activates the LC-NE system, many effects of VNS are blocked if the LC-NE system is impaired through either LC lesion or adrenergic receptor blockers. For example, the anticonvulsive effect of VNS is abrogated when hippocampal adrenergic receptors are blocked (Krahl et al., 1998; Raedt et al., 2011). Further, VNS enhancement of perforant path-CA3 synaptic transmission is blocked by either electrical lesions of the LC or an adrenergic receptor antagonist (timolol) (Shen et al., 2012). The antidepressant-like effects of VNS in rats, as measured by feeding and swim tests, have been shown to be blocked by lesion of noradrenergic neurons (Furmaga et al., 2011; Grimonprez et al., 2015). Immunotoxin depletion of norepinephrine was also found to prevent VNS-driven enhancement of motor cortex neuroplasticity (Hulsey et al., 2019).

### Vagus Nerve Stimulation and Cholinergic Systems

Cholinergic nuclei of the basal forebrain (BF) project to the sensory processing regions of the thalamus (Kolmac and Mitrofanis, 1999) and cortex (Ballinger et al., 2016; Jimenez-Martin et al., 2021). Additionally, cholinergic nuclei of the pontomesencephalic area, including the laterodorsal tegmental nucleus (LDT) and pedunculopontine tegmental nucleus (PPT), are a major source of ACh to the thalamus (Schofield et al., 2011; Huerta-Ocampo et al., 2020). There are two distinct neuron populations of the BF that differentiate in exhibiting either a tonic (10–15 Hz) or a bursting (2–6 spikes/burst with bursting events occurring at 0.3–2 Hz) firing pattern (Nuñez, 1996) which influences arousal and attention. The response timing of both types of BF neurons is influenced by sensory stimuli (Laszlovszky et al., 2020) and linked with novelty, salience, and surprise (Zhang et al., 2019).

Extensive work has shown the cholinergic system strongly influences both sensory processing and perceptual learning across multiple sensory modalities (Murphy and Sillito, 1991; Kilgard and Merzenich, 1998; Verdier and Dykes, 2001; Linster and Cleland, 2002; Bentley et al., 2004; Wilson et al., 2004; Furey et al., 2008; Herrero et al., 2008; Pinto et al., 2013; Zhan et al., 2013; Rothermel et al., 2014; Kim et al., 2016; Gratton et al., 2017). Like the noradrenergic system, it is well documented that activation of the cholinergic systems has immediate effects on sensory processing. ACh applied *in vitro* to neurons of the thalamic reticular nucleus, a subthalamic region involved in sensory processing, causes hyperpolarization and induces burst spiking (McCormick and Prince, 1986), likely due to extended hyperpolarized periods priming the calcium T-type channels responsible for burst spiking (Sherman, 2001a). ACh applied to thalamic neurons of the primary visual and auditory pathways was found to increase firing rate (Sillito et al., 1983; McCormick and Prince, 1987), although a hyperpolarization effect has been observed in thalamic neurons of the secondary (non-lemniscal) auditory pathway (Mooney et al., 2004). Cholinergic modulation of the sensory cortex can cause either facilitation or inhibition with the resulting effect specific to the sensory modality, cell, and stimulation pattern (Donoghue and Carroll, 1987; Metherate and Weinberger, 1989; Metherate and Ashe, 1991; Jimenez-Martin et al., 2021). In the visual cortex, BF stimulation has been shown to enhance accurate encoding by inducing decorrelation and increased reliability (Goard and Dan, 2009).

It has long been hypothesized that VNS activates the BF–ACh system (Detari et al., 1983). VNS innervates the NTS (Ruffoli et al., 2011) and projections from the NTS activate the BF (Martin et al., 2022) in addition to the NTS projections that activate the LC (Ennis and Aston-Jones, 1988; Van Bockstaele et al., 1999; Reyes and Van Bockstaele, 2006). The LC also projects to the BF (Berridge et al., 2003), suggesting VNS activates the BF both directly through the NTS as well as indirectly through the LC. Indeed, two separate studies investigating the potential of VNS for inducing neuroprotection from cerebral ischemia found that VNS enhanced protein levels of the nicotinic acetylcholine receptor alpha7 subunit (a7nAchR) in the ischemic penumbra (Jiang et al., 2014; Lu et al., 2017). Recently, researchers performed *in vivo* calcium imaging of the auditory cortex and found VNS evoked activity of cholinergic axons innervating the region (Mridha et al., 2021). Further, they found the intensity of the evoked activity covaried with VNS intensity. In addition to this direct evidence that VNS rapidly activates the cholinergic system, multiple studies have shown ACh modulation of sensory pathways is a critical component underlying the plasticity effect induced by repeatedly pairing a burst of VNS with a sensory stimulus. For example, the effects of VNS on sensory processing in the auditory cortex were found to be blocked by a muscarinic antagonist (Nichols et al., 2011). Further, lesioning the NB in rats was shown to abrogate the well-documented ability of VNS pulses repeatedly paired with a movement to enhance motor cortex plasticity (Hulsey et al., 2016).

### Vagus Nerve Stimulation and Serotonergic Systems

The dorsal raphe nucleus (DRN) is a major source of serotonin (5-HT) to the forebrain (Jacobs and Fornal, 1999). Neurons of the DRN consistently exhibit a continuous slow tonic firing rate (1–2 Hz) with little variation in inter-spike-interval (Trulson and Jacobs, 1979; Mlinar et al., 2016). Response of the DRN is related to both reward and punishment (Ranade and Mainen, 2009; Li et al., 2016; Ren et al., 2018) as well as linked to sensory input (Rasmussen et al., 1984; Waterhouse et al., 2004). The DRN innervates both cortical and subcortical regions of the sensory processing pathways (Kirifides et al., 2001). There is also a large body of work suggesting DRN activity modulates sensory processing and perception (Hurley and Pollak, 1999; Kähkönen et al., 2002; Dacks et al., 2009; Hurley and Hall, 2011; Jaber et al., 2014; Kapoor et al., 2016; Seillier et al., 2017). 5-HT has been shown to have instant effects on neurons of the sensory pathways. For example, 5-HT has been shown to cause excitation of thalamic perigeniculate and reticular nucleus neurons (McCormick and Wang, 1991; Funke and Eysel, 1993). In the inferior colliculus, an auditory region of the midbrain, 5-HT was found to modulate responses in both a cell and auditory stimulus specific manner (Hurley and Pollak, 1999). In the primary visual and auditory relay neurons of the visual and auditory pathways, 5-HT has been shown to have an inhibitory effect (Marks et al., 1987; Kayama et al., 1989; Monckton and McCormick, 2002). Additionally, activation of the DRN has been found to increase signal to noise ratio of the olfactory cortex (Lottem et al., 2016).

Vagus nerve stimulation may activate the DRN indirectly by first activating the LC which then projects to the DRN (Kim et al., 2004). This hypothesis is supported by a study in rats anesthetized with sodium pentobarbital that found VNS increased DRN neurons’ firing rates, but this causal relationship was lost once the LC was lesioned (Manta et al., 2009a). Multiple studies have also shown that VNS increases DRN firing rate as measured *via* extracellular electrophysiological recordings (Dorr and Debonnel, 2006; Manta et al., 2009b). However, one study found only a subset of VNS patterns they tested increased DRN activity suggesting VNS activation of the DRN may be dependent on VNS parameters (Manta et al., 2012). In a follow-up work, the same group performed *in vivo* microdialysis in rats following chronic duty-cycled VNS and found increased 5-HT concentration in the DRN but not the hippocampus nor prefrontal cortex (PFC) (Manta et al., 2013). In contrast to these studies supporting VNS’ ability to activate the DRN, another study analyzing microdialysis measurements in different brain regions of rats reported that neither vagotomy or chronic unilateral VNS had an effect on 5-HT levels in the ventral tegmental area (VTA), nucleus accumbens (NAc), PFC, and striatum (Ziomber et al., 2012). These conflicting findings could potentially be related to the fact that electrical stimulation was delivered to an abdominal branch of the vagus nerve in this study. Further suggesting a more complex interplay between the VNS and DRN, a study analyzing immunohistochemical biomarkers of both short-term and long-term neuronal activation suggests chronic VNS does not induce DRN activation until stimulation has occurred across multiple days (Cunningham et al., 2008).

In addition to direct evidence that VNS increases activity of the serotonergic system, functionality of serotonergic neurons has been shown to be critical for multiple documented effects of VNS. For example, the earlier-mentioned study on the antidepressant-like effects of VNS in rats, which used feeding and swim tests as indexes of depression, found the beneficial effects of VNS were also precluded by administration of a neurotoxin for serotonergic neurons (Furmaga et al., 2011). Additionally, a separate study found immunotoxin depletion of serotonin prevented the well-researched ability of repeatedly pairing a VNS burst with a movement to enhance motor cortex neuroplasticity (Hulsey et al., 2019).

### Vagus Nerve Stimulation and Dopaminergic Systems

The VTA and Substantia Nigra pars Compacta (SNc) are primary sources of dopamine (DA) to the forebrain (Poulin et al., 2018) and, respectively, they modulate cognition and movement (Mercuri et al., 1992). The VTA has been shown to innervate the sensory cortices (Hosp et al., 2019). The VTA exhibits both tonic (1–8 Hz) and burst firing (2–5 spike bursts with bursting events occurring at 0.1–1 Hz) with firing rates varying across cell types (Kiyatkin and Rebec, 1998; Hyland et al., 2002; Lodge and Grace, 2006). Tonic firing rate likely modulates brain state (e.g., motivation and arousal) and bursting events likely encode for salient stimuli (e.g., reward and sensory stimuli) (Dahan et al., 2007). Although the body of work investigating the effects of DA on sensory processing is limited, there is evidence it rapidly modulates sensory processing and response (Ungless, 2004; Govindaiah et al., 2010; Woolrych et al., 2021).

Although previous work demonstrated the LC projects to the VTA (Mejías-Aponte et al., 2009), many studies also suggest VNS effects on DA circuitry may be dependent on other factors besides VNS directly increasing VTA firing rates. For example, one study that performed *in vivo* microdialysis of rats following chronic duty-cycled VNS found an increase in DA in the PFC and NAc but a decrease in VTA neurons’ firing rates as measured with electrophysiological recordings (Manta et al., 2013). A lack of VNS-induced changes in VTA firing and bursting rates was also reported in a separate study (Perez et al., 2014). Studies analyzing brain sections from rats that received chronic VNS have also reported varied results. One such study found decreased DA levels in the VTA, NAc, PFC, and striatum (Ziomber et al., 2012); however, to properly interpret these results it should be mentioned that electrical stimulation was delivered to an abdominal branch of the vagus nerve in this study. Two other studies performing a similar analysis found VNS induced changes to the elemental composition of dopamine-related brain structures (Szczerbowska-Boruchowska et al., 2012) and to the lipids and proteins within the VTA, NAc, SNc, striatum, dorsal motor nucleus of vagus, and the motor cortex (Surowka et al., 2015). A more recent study in awake rats found optogenetic VNS, which carries no risk of unintentional activation of surrounding nerves, increased the firing rate of dopaminergic VTA neurons as measured *via in vivo* imaging (Fernandes et al., 2020). This same study also found lesioning the hepatic branch of the vagus nerve abrogated the increase in VTA neuron activity usually observed following ingestion.

## Discussion: Translational Applications of Using Vagus Nerve Stimulation to Rapidly Modulate Sensory Processing

Accurate perception is required for daily life and independent living. However, dysfunction or degradation of central sensory processing pathways can rapidly impair sensory ability. The studies referenced here implicate VNS as a potential tool for modulating sensory processing. Accordingly, VNS presents great potential as a targeted treatment for impaired senses arising from central sensory processing dysfunction. Many clinical causes of impaired central sensory processing exist including multiple neurodegenerative conditions and neurological disorders. Impaired sensory processing reduces sensory acuity, increases likelihood of miscommunication, and causes misperceptions that potentially lead to costly human error. Further, the link between human performance and sensory ability suggests there may be commercial interest in enhancing sensory processing in addition to clinical. This translation potential has spurred clinical trials looking at the effect of VNS on auditory perception (e.g., NCT04812015 at www.clinicaltrials.gov). VNS methods of enhancing sensory processing have great translation possibility because cervical transcutaneous VNS (ctVNS) and taVNS have both been suggested to be safe and effective methods of non-invasively activating the vagus nerve in humans (Frangos and Komisaruk, 2017; Mwamburi et al., 2017; Reuter et al., 2019; McIntire et al., 2021). In light of this potential, our research team is currently conducting pilot clinical studies investigating the effects of continuous tonic VNS on vision, hearing, and touch.

Age-related impairment of central sensory processing is particularly devastating to the elderly as it interferes with their ability to communicate (Tun et al., 2012; Sardone et al., 2019), accelerates cognitive decline (Hewitt, 2017), and is linked with Alzheimer’s disease (AD) (Panza et al., 2015). Treatments exist for age-related sensory receptor damage (Barriga-Rivera et al., 2017; Ferguson et al., 2017; Higuchi et al., 2017; Moshirfar et al., 2017). However, there is a stark lack of solutions addressing the co-occurring age-related impairment of central sensory processing (Humes et al., 2013; Humes, 2015; Engel-Yeger and Rosenblum, 2017; Lesica, 2018). For example, as evidence of this age-related decline in sensory processing, studies have shown that elderly individuals with normal audiograms, indicating normally functioning auditory receptors, still have decreased ability to discriminate detailed features of sensory stimuli, such as speech intelligibility over noise (Fullgrabe et al., 2014; Babkoff and Fostick, 2017). Similarly, aging is thought to degrade visual (Brannan, 1992; Wiegand et al., 2014) and tactile processing (Engel-Yeger et al., 2012). The ability to improve or restore sensory processing clarity with VNS, could therefore positively impact a large segment of society by helping them remain social and active through improving their ability to communicate clearly and walk safely. Many researchers share the belief that different forms of VNS could help elderly cognition and perception as suggested by the many ongoing clinical studies investigating that topic (e.g., Clinical Trials NCT04396249, NCT04276805, NCT03359902, NCT04908358, NCT04276805, and NCT03989375 at www.clinicaltrials.gov).

Attention deficit hyperactivity disorder (ADHD) has been linked with impaired sensory processing evidenced by poor frequency discrimination ability (Sutcliffe et al., 2006; Shimizu et al., 2014). Moreover, inattention is linked with increased bursting activity in the sensory thalamus, a type of neural activity our team’s research has found is suboptimal for encoding details and features of sensory stimuli therefore causing loss of sensory acuity (Rodenkirch et al., 2019). Further, thalamocortical bursting in response to sensory stimuli is thought to serve as a “wake-up-call” in response to salient stimuli, suggesting bursts are distracting (Sherman, 2001b). Recently, poor intrathalamic processing due to abnormal TRN responses has been suggested as a cause of ADHD (Wells et al., 2016). ADHD treatments (including stimulants) work, in part, *via* amplifying NE effects (Arnsten and Dudley, 2005; De Crescenzo et al., 2018; Schneider et al., 2019). Methylphenidate, a common treatment for ADHD, has been shown to enhance early-stage sensory processing through increasing DA and NE concentration in the brain (Navarra et al., 2017). Previous work shows that VNS activates the locus coeruleus-norepinephrine (LC-NE) system (Hulsey et al., 2017), and our work shows VNS suppresses noisy bursting activity along sensory pathways. Taken together, these findings suggest VNS could be potentially used to treat the sensory processing dysfunction linked with ADHD.

## Author Contributions

CR and QW wrote the manuscript with input from JC. All authors contributed to the article and approved the submitted version.

## Conflict of Interest

All authors have financial interest in Sharper Sense, a company developing methods of enhancing sensory processing with VNS.

## Publisher’s Note

All claims expressed in this article are solely those of the authors and do not necessarily represent those of their affiliated organizations, or those of the publisher, the editors and the reviewers. Any product that may be evaluated in this article, or claim that may be made by its manufacturer, is not guaranteed or endorsed by the publisher.
